# Supplementary material for: Dynamic states of population activity in prefrontal cortical networks of freely-moving macaque
Source: Nat Commun. 2020 Apr 23;11:1948. doi: 10.1038/s41467-020-15803-x (PMC7181779; doi:10.1038/s41467-020-15803-x)
Supplement: Supplementary file 1 — Supplementary Information [file 41467_2020_15803_MOESM1_ESM.pdf]

Supplementary Information

Dynamic states of population activity in prefrontal cortical networks of freely-moving macaque

Milton et al.

Supplementary Figures

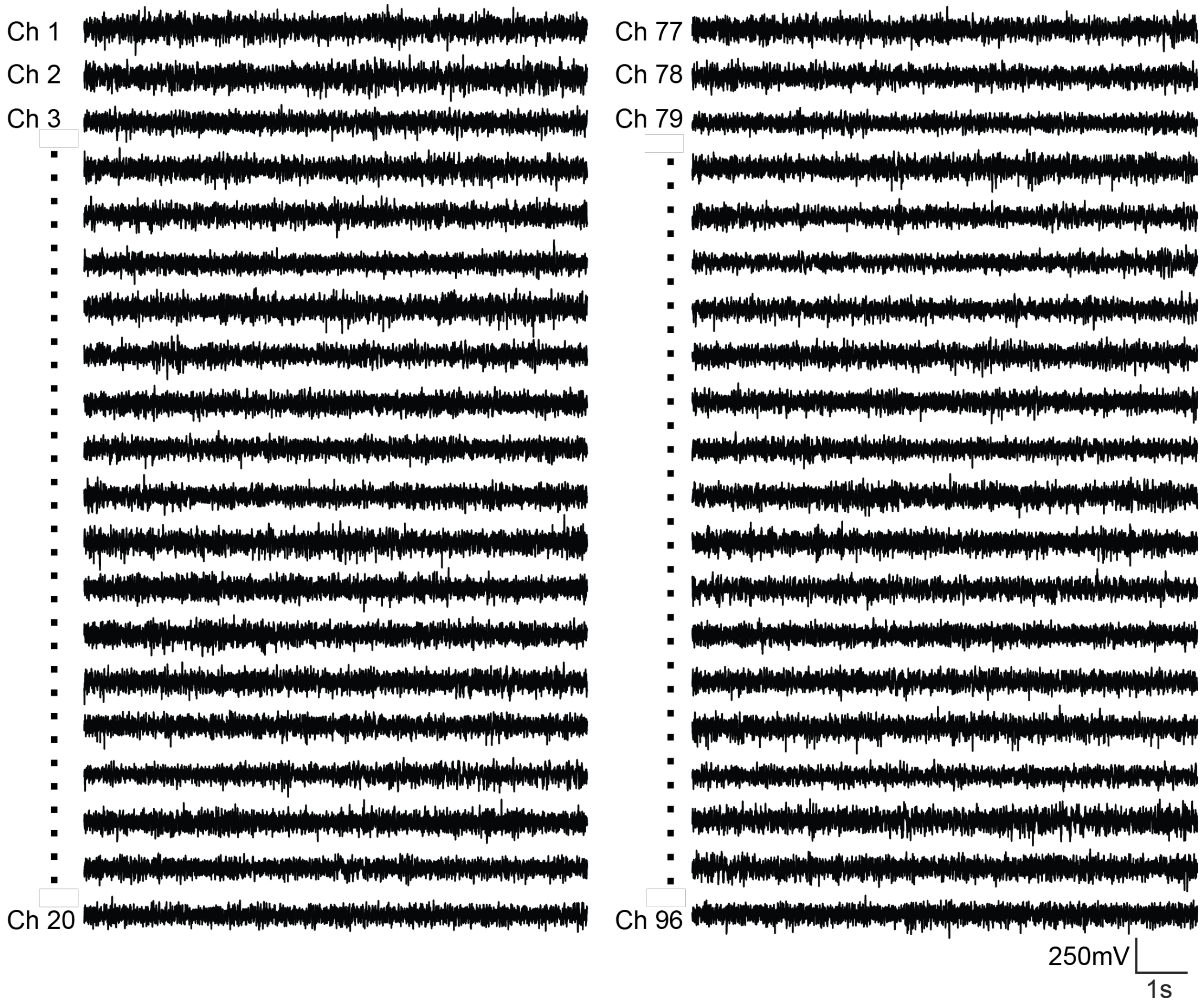

Supplementary Figure 1 | Example of wireless electrophysiological recordings in dorsolateral prefrontal cortex of freely-moving macaque. Each trace represents 2 kHz local field potential recordings from the 96-channel array during the same 10s epoch. Each channel presented has at least one well-isolated single unit after offline spike sorting.

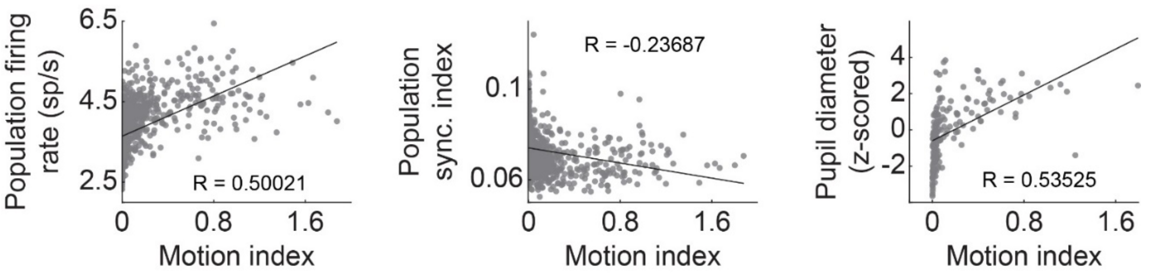

Supplementary Figure 2 | Single session relationship with motion index of population firing rate, PSI, and pupil diameter.
(**left**) Significant positive correlation between motion index and population firing rate in an example session (Pearson's correlation, $R = 0.3890$, $p < 0.0001$, $n = 912$ epochs).
(**middle**) Significant negative correlation between motion index and PSI in an example session (Pearson's correlation, $R = -0.2217$, $p < 0.05$, $n = 912$ epochs).
(**right**) Significant positive correlation between motion index and z-scored pupil diameter in an example session (Pearson's correlation, $R = 0.5352$, $p < 0.05$, $n = 912$ epochs).

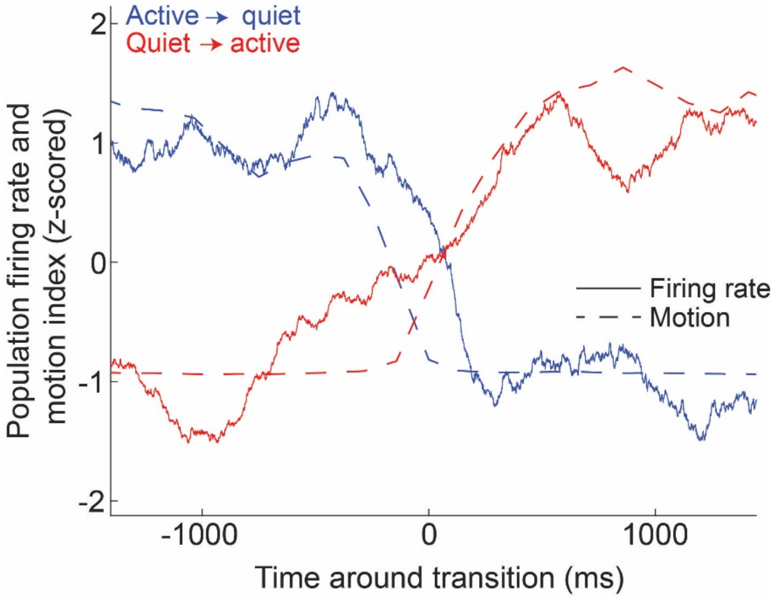

Supplementary Figure 3 | Single session example shows transitions between active and quiet wakefulness closely track with changes in population firing rate. Motion index and population firing rate were both aligned to transition times and z-scored for comparison. Active to quiet transitions are shown in blue, and quiet to active transitions are shown in red. The solid lines are the average population firing rate for all transitions, and the dashed lines are the average motion index for all transitions.

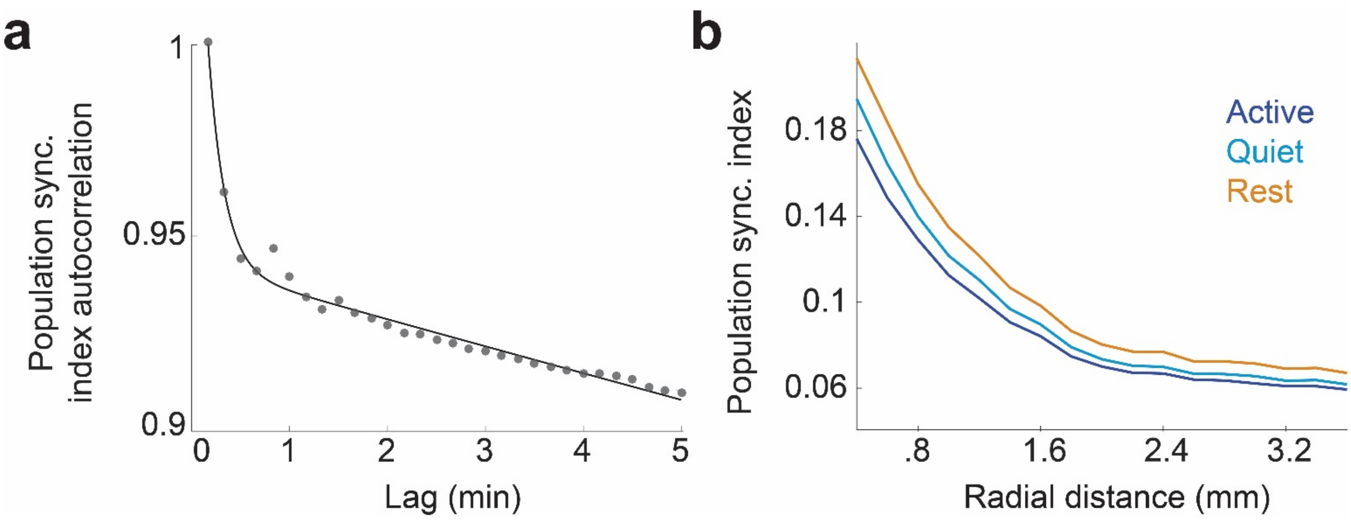

Supplementary Figure 4 | Spatial and temporal persistence of population synchrony index.
(**a**) Average autocorrelation of population synchrony index over time for all sessions. Autocorrelation remains over 90% at a 5-min lag, demonstrating that synchrony changes over long timescales. Grey dots indicate the average autocorrelation of PSI computed from the data, black line is a fitted exponential trend line.
(**b**) Population synchrony index computed for subpopulations within a given radial distance. At each radial distance, a significant difference was observed between active, quiet, and rest states (Friedman test, $p < 0.001$; 2-sided Wilcoxon rank-sum test, $p < 0.05$). The relationship between population synchrony and distance demonstrates that local populations are more synchronized than more distant populations, and that the difference in synchrony across behavioral states persists across all spatial scales accessible in these experiments.

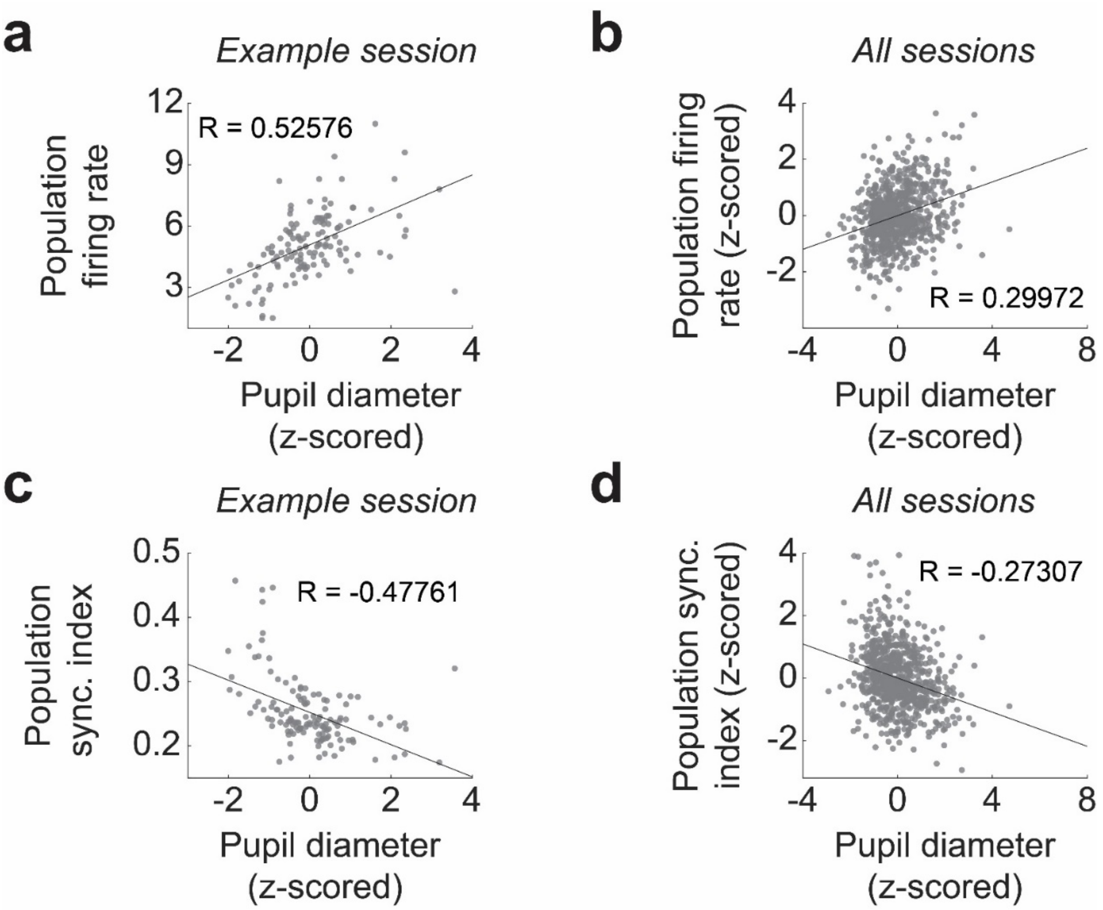

Supplementary Figure 5 | Pupil diameter reflects cortical state in freely-moving monkey.
(**a**) Example session showing a significant correlation between z-scored pupil diameter and population firing rate (Pearson's correlation, R = 0.52576, p < 0.0001, n = 129 epochs).
(**b**) When combined across sessions, there is a significant correlation between z-scored pupil diameter and z-scored population firing rate (Pearson's correlation, R = 0.29972, p < 0.0001, n = 2164 epochs).
(**c**) Example session showing a significant negative correlation between z-scored pupil diameter and PSI (Pearson's correlation, R = -0.47761, p < 0.0001, n = 129 epochs).
(**d**) When combined across all sessions, there is a significant negative correlation between z-scored pupil diameter and z-scored PSI (Pearson's correlation, R = -0.27307, p < 0.0001, n = 2164).

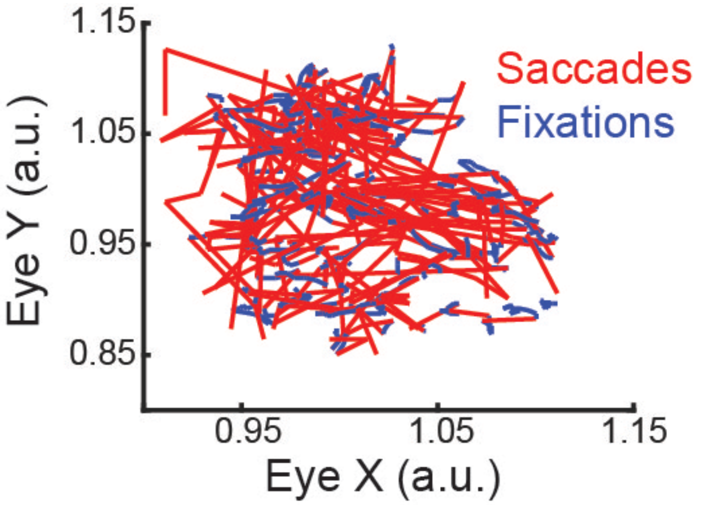

Supplementary Figure 6 | Example eye position trace showing saccades and fixations recorded wirelessly.

Horizontal and vertical coordinates of the pupil were recorded and used to compute eye velocity. Eye velocity was thresholded at one standard deviation above the median to extract saccades and fixations. Any period of time greater than 5ms during which the eye was below this threshold was considered a fixation.

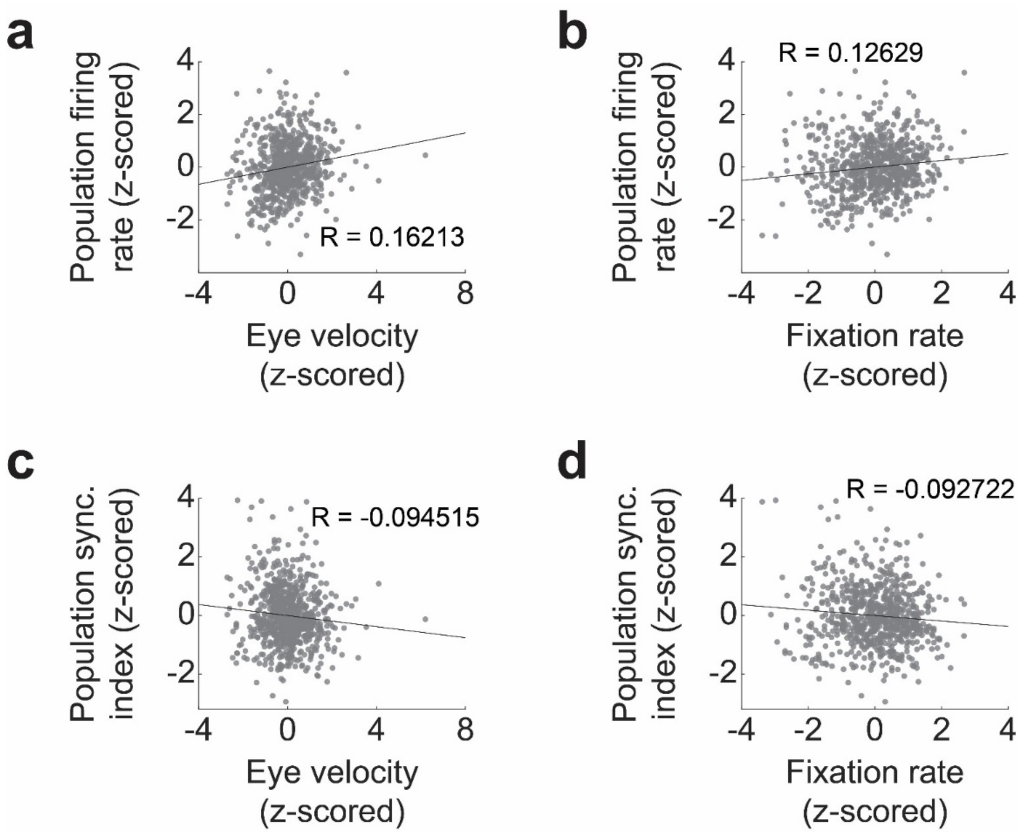

Supplementary Figure 7 | Cortical state is weakly correlated with eye movements.
(**a**) When combined across sessions and z-scored, population firing rate is positively correlated with eye velocity (Pearson's correlation, $R = 0.16213$, $p < 0.0001$, $n = 2164$).
(**b**) Population firing rate and fixation rate are positively correlated when combined across sessions (Pearson's correlation, $R = 0.12629$, $p < 0.001$, $n = 2164$).
(**c**) When combined across sessions and z-scored, PSI is not significantly correlated with eye velocity (Pearson's correlation, $p > 0.1$, $n = 2164$).
(**d**) PSI is not significantly correlated with fixation rate when combined across sessions (Pearson's correlation, $p >0.1$, $n = 2164$).

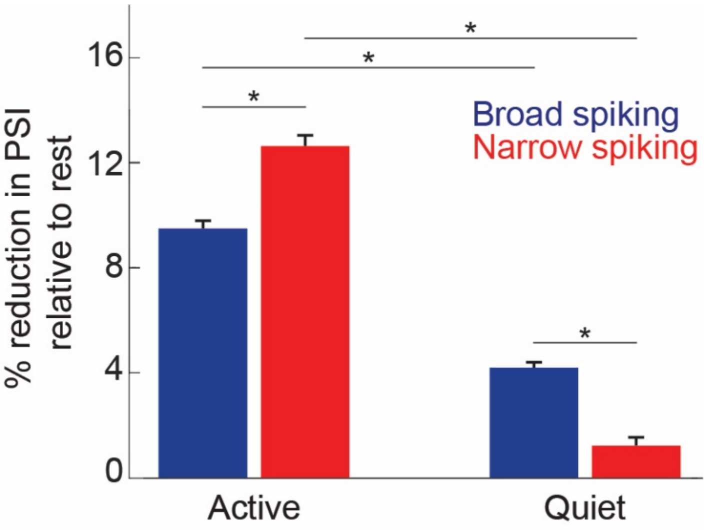

Supplementary Figure 8 | Differential modulation of broad-spiking and narrow-spiking subpopulation synchrony across behavioral states. Population synchrony index was computed for broad-spiking and narrow-spiking subpopulations across all behavioral states, and the percent difference from rest is shown. Significant differences across all groups were confirmed (Kruskal-Wallis test, $p < 0.0001$). Each subpopulation in each wake state was significantly different from rest value (Wilcoxon rank-sum test with Bonferroni correction, $p < 0.001$). Both subpopulations were significantly desynchronized in the active state relative to the quiet state (Wilcoxon rank-sum test with Bonferroni correction, $p < 0.0001$). The narrow-spiking subpopulation was more desynchronized in the active state relative to the broad-spiking subpopulation (Wilcoxon rank-sum test with Bonferroni correction, $p < 0.0001$). During the quiet state, the narrow-spiking population was closer to its rest value than the broad-spiking population (Wilcoxon rank-sum test with Bonferroni correction, $p < 0.0001$; error bars indicate mean +/- standard error).
