# Supplementary material for: Dynamic states of population activity in prefrontal cortical networks of freely-moving macaque
Source: Nat Commun. 2020 Apr 23;11:1948. doi: 10.1038/s41467-020-15803-x (PMC7181779; doi:10.1038/s41467-020-15803-x)
Supplement: Supplementary file 3 — Reporting Summary [file 41467_2020_15803_MOESM3_ESM.pdf]

nature research

Corresponding author(s): Valentin Dragoi

Last updated by author(s): Mar 10, 2020

Reporting Summary

Nature Research wishes to improve the reproducibility of the work that we publish. This form provides structure for consistency and transparency in reporting. For further information on Nature Research policies, see Authors & Referees and the Editorial Policy Checklist.

Statistics

For all statistical analyses, confirm that the following items are present in the figure legend, table legend, main text, or Methods section.

n/a	Confirmed	
☐	☒	The exact sample size (n) for each experimental group/condition, given as a discrete number and unit of measurement
☒	☐	A statement on whether measurements were taken from distinct samples or whether the same sample was measured repeatedly
☐	☒	The statistical test(s) used AND whether they are one- or two-sided<br>*Only common tests should be described solely by name; describe more complex techniques in the Methods section.*
☒	☐	A description of all covariates tested
☐	☒	A description of any assumptions or corrections, such as tests of normality and adjustment for multiple comparisons
☐	☒	A full description of the statistical parameters including central tendency (e.g. means) or other basic estimates (e.g. regression coefficient) AND variation (e.g. standard deviation) or associated estimates of uncertainty (e.g. confidence intervals)
☐	☒	For null hypothesis testing, the test statistic (e.g. F, t, r) with confidence intervals, effect sizes, degrees of freedom and P value noted<br>*Give P values as exact values whenever suitable.*
☒	☐	For Bayesian analysis, information on the choice of priors and Markov chain Monte Carlo settings
☒	☐	For hierarchical and complex designs, identification of the appropriate level for tests and full reporting of outcomes
☐	☒	Estimates of effect sizes (e.g. Cohen's d, Pearson's r), indicating how they were calculated

Our web collection on statistics for biologists contains articles on many of the points above.

Software and code

Policy information about availability of computer code

Data collection	We collected and synchronized data across three separate streams. Neural data was recorded on Blackrock Microsystems hardware using their Central suite. Wireless eye tracking used Iscan's eye tracking software. Videography used the OpenCV3 package in python3.
Data analysis	Data analysis used MATLAB (including the Chronux package for LFP analysis). RWA-Web was used for relative weight analysis of multiple regression (24). OpenCV3 package was used in video processing.

For manuscripts utilizing custom algorithms or software that are central to the research but not yet described in published literature, software must be made available to editors/reviewers. We strongly encourage code deposition in a community repository (e.g. GitHub). See the Nature Research guidelines for submitting code & software for further information.

Data

Policy information about availability of data

All manuscripts must include a data availability statement. This statement should provide the following information, where applicable:

- Accession codes, unique identifiers, or web links for publicly available datasets
- A list of figures that have associated raw data
- A description of any restrictions on data availability

The data used in this study are available from the corresponding author upon reasonable request.

Field-specific reporting

Please select the one below that is the best fit for your research. If you are not sure, read the appropriate sections before making your selection.

☒ Life sciences ☐ Behavioural & social sciences ☐ Ecological, evolutionary & environmental sciences

Life sciences study design

All studies must disclose on these points even when the disclosure is negative.

Sample size	Sample size consisted of number of recording sessions from 2 monkeys, number of single units, or number of 10-second epochs recorded throughout all sessions. Sample size was not predetermined, but is consistent with similar studies.
Data exclusions	No data was excluded.
Replication	We tested the repeatability of our initial findings of behavioral state-dependent population synchrony in dlPFC by demonstrating that these results are significant when analyzed within individual sessions, such that each session (11 total) would represent a replication.
Randomization	In this study, experimental groups correspond to naturally occurring behavioral states, such that every animal contributes data to all groups at different times, based on the intrinsic randomness of their own behavior. There were no extrinsic task variables to randomize.
Blinding	Blinding was not necessary as all animals were recorded in all behavioral states.

Reporting for specific materials, systems and methods

We require information from authors about some types of materials, experimental systems and methods used in many studies. Here, indicate whether each material, system or method listed is relevant to your study. If you are not sure if a list item applies to your research, read the appropriate section before selecting a response.

Materials & experimental systems

n/a	Involved in the study	
☒	☐	Antibodies
☒	☐	Eukaryotic cell lines
☒	☐	Palaeontology
☐	☒	Animals and other organisms
☒	☐	Human research participants
☒	☐	Clinical data

Methods

n/a	Involved in the study	
☒	☐	ChIP-seq
☒	☐	Flow cytometry
☒	☐	MRI-based neuroimaging

Animals and other organisms

Policy information about studies involving animals; ARRIVE guidelines recommended for reporting animal research

Laboratory animals	Two male rhesus monkeys (Macaca mulatta; both 9 years old)
Wild animals	No wild animals were used.
Field-collected samples	No field-collected samples were used.
Ethics oversight	All experiments were performed in accordance with protocols approved by the Animal Welfare Committee (AWC) and the Institutional Animal Care and Use Committee (IACUC) for the University of Texas Health Science Center at Houston (UTHealth).

Note that full information on the approval of the study protocol must also be provided in the manuscript.
